# Development of a micro-tissue-mediated injectable bone tissue engineering strategy for large segmental bone defect treatment

**DOI:** 10.1186/s13287-018-1064-1

**Published:** 2018-11-28

**Authors:** Dingyu Wu, Zhenxing Wang, Jinbing Wang, Yingnan Geng, Zhanzhao Zhang, Yu Li, Qiannan Li, Zhiwei Zheng, Yilin Cao, Zhi-Yong Zhang

**Affiliations:** 1 Department of Plastic and Reconstructive Surgery, Shanghai 9th People’s Hospital, Shanghai Key Laboratory of Tissue Engineering, School of Medicine, Shanghai Jiao Tong University, Shanghai, 200011 China; 2 Translational Research Centre of Regenerative Medicine and 3D Printing Technologies of Guangzhou Medical University, The Third Affiliated Hospital of Guangzhou Medical University, No.63 Duobao Road, Liwan District, Guangzhou City, 510150 Guangdong Province China; 3 China Orthopedic Regenerative Medicine Group (CORMed), Hangzhou, 310058 China; 4 Department of Plastic Surgery, Union Hospital, Tongji Medical College, Huazhong University of Science and Technology, Wuhan, 430022 China

**Keywords:** iBTE, Micro-tissue, Large segmental bone defect, BMSCs, Bone regeneration

## Abstract

**Background:**

Bone tissue engineering is not widely used in clinical treatment. Two main reasons hide behind this: (1) the seed cells are difficult to obtain and (2) the process of tissue engineering bone construction is too complex and its efficiency is still relatively low. It is foreseeable that in the near future, the problem of seed cell sources could be solved completely in tissue engineering bone repair. As for the complex process and low efficiency of tissue engineering bone construction, usually two strategies would be considered: (1) the construction strategy based on injectable bone tissue and (2) the construction strategy based on osteogenic cell sheets. However, the application of injectable bone tissue engineering (iBTE) strategy and osteogenic cell sheet strategy is limited and they could hardly be used directly in repairing defects of large segmental bone, especially load-bearing bone.

**Methods:**

In this study, we built an osteogenic micro-tissue with simple construction but with a certain structure and composition. Based on this, we established a new iBTE repair strategy—osteogenic micro-tissue in situ repair strategy, mainly targeting at solving the problem of large segmental bone defect. The steps are as follows: (1) Build the biodegradable three-dimensional scaffold based on the size of the defect site with 3D printing rapid prototyping technology. (2) Implant the three-dimensional scaffold into the defect site. This scaffold is considered as the “steel framework” that could provide both mechanical support and space for bone tissue growth. (3) Inject the osteogenic micro-tissue (i.e., the “cell-extracellular matrix” complex), which could be considered as “concrete,” into the three-dimensional scaffold, to promote the bone tissue regeneration in situ. Meanwhile, the digested cells were injected as the compared group in this experiment. After 3 months, the effect of in situ bone defect repair of osteogenic micro-tissue and digested cells was compared.

**Results:**

It is confirmed that osteogenic micro-tissue could achieve a higher efficiency on cell usage and has a better repair effect than the digested cells.

**Conclusions:**

Osteogenic micro-tissue repairing strategy would be a more promising clinical strategy to solve the problem of large segmental bone defect.

**Electronic supplementary material:**

The online version of this article (10.1186/s13287-018-1064-1) contains supplementary material, which is available to authorized users.

## Background

For orthopedic doctors, the large segmental bone defect caused by trauma and tumor resection has always been a difficult problem in clinic. Many studies have confirmed that bone tissue engineering repair strategy is expected to solve this problem [1]. However, at present, the classical bone tissue engineering repair strategy has been widely proved in the experiment, but it is not widely used in clinical treatment. Two main reasons hide behind this: (1) the seed cells are difficult to obtain and (2) the process of tissue engineering bone construction is too complex and its efficiency is still relatively low.

Many researches have already been performed to solve the problem of seed cell obtainment from various approaches: (1) using bioreactors to proliferate the bone marrow mesenchymal stem cells in large scale in order to provide more seed cells for bone tissue engineering repair [2]; (2) many studies have clearly shown that non-bone tissue-derived mesenchymal stem cells, such as adipose-derived mesenchymal stem cells, umbilical cord-derived mesenchymal stem cells, and placental-derived mesenchymal stem cells, have similar osteogenic activity as the bone marrow-derived mesenchymal stem cells; thus, they could also be used as seed cells for bone tissue engineering [3]; (3) some scholars have studied the application of induced pluripotent stem cell (iPSC) in bone tissue engineering and have recently made significant achievement, which provides a new clinical transformation solution for the seed cell source in tissue engineering repair [3]; (4) our previous studies have demonstrated that allogeneic fetal-derived mesenchymal stem cells have high proliferative capacity and high osteogenic differentiation ability with low immunogenicity and no tumorigenicity, which could be preserved in a seed cell bank as a type of general seed cells [4]. Therefore, it is foreseeable that in the near future, the problem of seed cell sources could be solved completely in tissue engineering bone repair.

As for the second difficulty, namely the complex process and low efficiency of tissue engineering bone construction, we have not seen many studies at present. Usually, two strategies would be considered: (1) the construction strategy based on injectable bone tissue and (2) the construction strategy based on osteogenic cell sheets.

In recent years, the construction strategy of injectable bone tissue with high efficiency and simple process has become a research hotspot. The traditional injectable tissue-engineered bone repair strategy, which based on digested cells, would inject directly the digested cells to repair defect [5–11]. It is relatively simple and homogeneously mixed and favors its clinical application. However, this strategy, due to the lack of mechanical support of the constructed cell-gel complex, is currently applied only to cavity defect treatment. In addition, enzymes are required to digest the cells, which would damage the activity of the cells [12]. What is more, the extracellular matrix (ECM) around the cells could be destroyed and removed during cell digestion with enzymes, thus compromising its potential defect healing capacity [13].

Another construction strategy, osteogenic cell sheets, could effectively retain the extracellular matrix, and it has been broadly studied in various branches of tissue engineering and regenerative medicine. Early in 1993, Okano et al. developed the cell sheet technology that allows cells to form one or more layers of dense sheet in a culture dish after direct stimulation on cells to secrete extracellular matrices [14]. At present, this strategy has already been verified in the research of bone tissue engineering repair. In 2013, Liu [15] repaired skull defects in SD rats by bone marrow mesenchymal stem cell sheets combined with calcined bone. The result showed that the skull defect could be completely repaired, and the repair effect was significantly better than single-cell suspension group. However, the osteogenic cell sheets obtained from the dish cultivation could hardly maintain certain form without scaffold support because of their weak strength and tenacity. And if implanted together with scaffold or gel, the cell sheets could hardly be mixed evenly. Therefore, the application of osteogenic cell sheet strategy is limited and it could hardly be used directly in repairing defects of large segmental bone, especially load-bearing bone.

To solve these problems, increase the treatment efficacy of iBTE strategy, and broaden its clinical indication, we have developed a new iBTE strategy for large segmental defect treatment, based on the osteogenic micro-tissue and 3D printing scaffolds. First, we used osteogenic cell sheet fragmentation technology to develop osteogenic micro-tissue, which contains rich cells and ECM. This kind of osteogenic micro-tissue is simple, is efficient, and preserves extracellular matrix. These fragmented cell sheets could, on the one hand, curl or fold to form a tiny 3D structure, namely “micro-tissue,” and, on the other hand, allow a more even combination with scaffold or gel. Therefore, it could have a wider application than the intact cell sheets. It contains no other component except cells and extracellular matrix. Then, we combined the osteogenic micro-tissue with the 3D printing scaffold to construct a complex with reinforced concrete structure. This complex overcomes the shortcomings of the traditional iBTE and cell sheets and can be applied to repair large segmental defects, which demand high mechanical properties. In addition, in this strategy, 3D printing technology is used to customize the implanted scaffold, and we can accurately construct the implant complex according to the shape and size of the defect. Although scaffolds with good osteoinductivity were reported in many studies, we would rather choose the blank polycaprolactone (PCL), which has no osteoinductivity, as the scaffolds. In this way, the influence of the material to the experiment was minimized, which allows a better comparison of the two strategies’ repair effects. According to our previous studies, the mechanical properties of PCL make it a good support scaffold for bone repair [16]. Moreover, PCL could be slowly degraded and finally decomposed into CO_2_ and water, so its degradation will not have any influence on the experimental result [17, 18]. Therefore, all this together make PCL an ideal scaffold for bone repair in this study.

In this study, we applied this new iBTE strategy to repair the large segmental bone defects and used digested cells as the compared group, and further discussion was made on the reasons that cause their remarkably different repairing effects.

## Materials and methods

### PCL constructs customized

The polycaprolactone (PCL) was obtained from Shenzhen Esun Industrial Co., Ltd., China. PCL constructs were fabricated with a filament diameter of 500 μm and channel size of 1000 μm, with a 0–60–120° lay-down pattern by fused deposition modeling (FDM) technology, and prepared by NaOH (5 M, 37 °C) treatment for 24 h, as previously described [19]. The PCL scaffold was sterilized with epoxy ethane, then the disinfected PCL scaffold was soaked in alcohol for 30 min and washed three times with PBS, and in the end, the scaffold was dried; after drying, the scaffolds were sputter-coated with gold (BAL-TEC, Philips, Eindhoven, the Netherlands) and examined finally by a scanning electron microscope (PhilipsXL-30, the Netherlands). The porosity and mechanical properties of the PCL scaffold were described in our previous works [19]. Based on previous preparations, we have found that the PCL scaffold of such size is more favorable for the uniform injection and fixation of the gel composites.

### Isolation and culture of BMSCs

Rabbit bone marrow stromal cells (BMSCs) were obtained from fetal rabbits (age 28 days) according to a previous method [19]: After the abdomen fetus was taken out by cesarean section, the femurs and tibias of the fetus were separated; then, the long bone marrow cavities were repeatedly aspirated with syringes until the cavities appeared white; after that, the fresh bone marrow tissue was seeded onto 10-cm culture dishes with 7 mL of low glucose DMEM (Hyclone, Logan, UT, USA) supplemented with 10% FBS (Hyclone) and 1% penicillin and streptomycin (Thermo Fisher Scientific, Waltham, MA, USA). The culture dishes were incubated in a humidified environment (5% CO_2_, 37 °C), and the culture medium was changed every 3 days [2]. When the cell confluence reached 90%, 0.25% trypsin/1 mM EDTA (Thermo Fisher Scientific) was used to digest the MSCs (mesenchymal stem cells) for the passage until the fourth generation (P4). When passaged to P4, the cells were partly digested, seeded in 96-well plates at 1000 cells/well, then placed in a conventional incubator and incubated with cck-8 for 3 h on days 1, 3, 5, 7, and 9. With a micro-plate reader, the OD values of per hole and control hole were measured at 450 nm wavelength, and the difference value of the two was the final OD value. BMSCs (passage 4) were passaged into 6-well plates at a density of 1 × 10^5^ cells/well: three wells were stained with Alizarin red S after 2 weeks of osteogenic induction (DMEM, 10%fetal bovine serum, 50 mg/L ascorbic acid, 10 mmol/L sodium β-glycerophosphate, 1 × 10^−7^ mol/L dexamethasone), three wells were stained with Oil red O after 3 weeks of lipid induction (DMEM, 10%fetal bovine serum, 5 μg/mL insulin, 200 μM indomethacin, 1 μM dexamethasone, and 0.5 mM 3-isobutyl-1-methylxanthine), and three wells were stained with Alice blue after 3 weeks of chondrogenic induction (DMEM, 10%fetal bovine serum, 0.1 μM dexamethasone, 0.17 mM ascorbic acid, 1 mM sodium pyruvate, 0.35 mM l-proline, 1% insulin-transferrin sodium-selenite, 1.25 mg/mL bovine serum albumin, 5.33 μg/mL linoleic acid, and 0.01 μg/mL transforming growth factor-β). The same staining was done in the control groups.

### Osteogenic differentiation of BMSCs and cell sheet construction

The P4 generation BMSCs were seeded on the culture dish at 1 × 10^4^ cells/cm^2^. After confluence, the BMSCs were cultured to cell sheets with dense structure in osteogenic medium (10% fetal bovine serum, 50 mg/L ascorbic acid, 10 mmol/L sodium β-glycerophosphate, 1 × 10^−7^ mol/L dexamethasone), which was changed every 3 days for 3 weeks. The frozen sections of the cell sheets were made and stained with H&E and Sirius red, as previously described [12, 19]. They were observed under a microscope.

### Osteogenic micro-tissue preparation and characterization

For one dish of the cells, the supernatant was aspirated and the cells were washed with PBS. Then, the digested cells were obtained by trypsin. The cells were stained by trypan blue staining and counted under an inverted microscope. The digested cells were considered as the compared group. After sucking up the supernatant of another dish of cells and cleaning the cell sheets with PBS, the cell sheets were gently fragmented into small pieces, namely “osteogenic micro-tissue,” with a sterile blade. The osteogenic micro-tissue was collected for frozen section and stained by H&E (hematoxylin and eosin staining), Masson, PAS (Periodic acid Schiff reaction), von Kossa respectively, as previously described [12, 19].

Cellular quantities of osteogenic micro-tissue, digested cells, and initial cell sheet were measured by quantifying the dsDNA content using a Picogreen dsDNA Quantification Kit (Molecular Probes, USA) (*N* = 4) as previously described [2]. Both of the osteogenic micro-tissue and the digested cell groups were respectively stained with trypan blue (0.4%) at room temperature. After 5 min, the dye solution was suck out, the residual stain was washed with PBS, and the blue-stained cells were observed under a light microscope.

Calcein-AM/PI cell staining was also performed on both groups: (1) Before staining, 10 μl of Calcein-AM stock solution and 15 μL of PI stock solution were added into 5 mL of PBS to prepare a staining solution. (2) The micro-tissue and digested cells were washed by PBS until completely clean, then 100 μL of staining solution was added in each dish, and the cells were incubated at 37 °C for 15 min. (3) The two groups of cells were observed under the laser confocal microscope: firstly to observe the yellow-green live cells under a wavelength of 490 ± 10 nm, then to observe the red dead cells with a wavelength of 545 nm. At least three non-overlapping fields were randomly taken from each dish, and the test was repeated for three times.

Western protein analysis was also performed as previously described [20]: Cells were lysed in RIPA with an inhibitor cocktail (Sigma); the concentration of the protein was measured by a BCA protein assay (Thermo Fisher Scientific); after separating on a Tris-glycine SDS-PAGE gel (Invitrogen), the protein was transferred onto a PVDF membrane, followed by blocking in 5% BSA for 1 h; membranes were incubated with primary antibody overnight at 48 °C, and then with secondary antibody at 20 °C for 1 h. Secondary antibodies were OCN (ab13418, abcam), BMP2 (ab6285, abcam), Col1A1 (NB600-450, NOVUS), DDK1(LS-C47394, LSBio), and ALP (NB600-540, NOVUS).

### Repair experiments of large segmental bone defects in situ

**Fig. 1:**
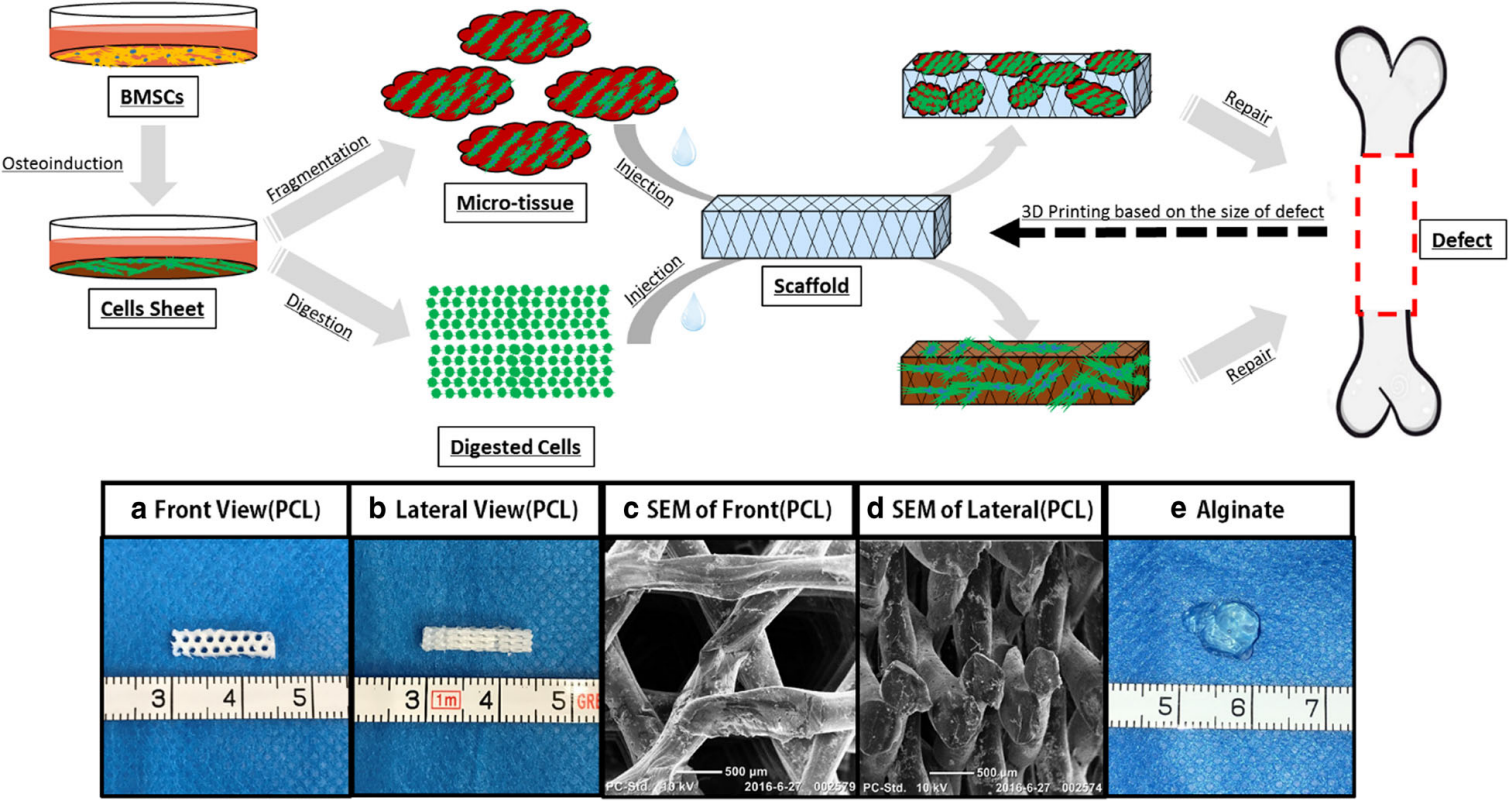
Schematic illustrations of the procedures for construction and the comparison of the micro-tissue and digested cells. **a**. General view of PCL scaffold (front); **b**. General view of PCL scaffold (side); **c**. An electron microscope of PCL scaffold (front); **d**. An electron microscope of PCL scaffold (side); **e**. General view of a calcium alginate gel

In strict accordance with the regulations of medical animal experiments, 12 New Zealand white rabbits (6 months, about 2.6 kg) were divided into osteogenic micro-tissue group, digested cell group, and blank group, each with 1.5% pentobarbital sodium (2.5 mL/kg) for anesthesia. The left anterior descending leg of the rabbits was skinned and disinfected. The skin and fascia were dissected from the radial face, and the vascular nerves were isolated. The radius was cut 30 mm away from the wrist joint, resulting in a defect of 15 mm. At the same time, the periosteum of 5 mm was stripped off the upper and lower segments of the radius, and the broken ends of the radius defect were washed repeatedly with normal saline, so as to remove the residual periosteum tissue. In the osteogenic micro-tissue group, 500 μl of 1.5% sodium alginate solution was directly used to resuspend the small pieces of tissue, and after that, an equal volume of 30 mmol/L CaCl_2_ solution was added to prepare a cell-gel complex. In the digested cell group, the cell suspension was obtained and then mixed the cells with gel. In the repair experiment, we put the 3D-printed prefabricated 15 mm × 5 mm × 3 mm PCL porous scaffold into the defect (the volume of defect was about 310 mm^3^, the volume of scaffold was about 225 mm^3^), and then, the previously made osteogenic micro-tissue composite (experimental group), digested cell-gel composite (control group), and pure gel group (blank group) of 80 μl were injected into each group of PCL porous scaffolds. Finally, we fasted the fascia and skin aseptically and tightly and administered 40 W units of penicillin to the buttocks of each New Zealand white rabbit for 3 days postoperatively. Immediately after surgery, X-ray examination of the radial defect was performed. One month and 3 months after surgery, we anesthetized the rabbits and performed X-ray examination of the radial defect. Three months later, 12 New Zealand White rabbits were over-anesthetized and euthanized. Then, micro-CT was performed. After scanning, the specimens was reconstructed by three-dimensional reconstruction software (VG studio Volume Graphics GmbH, Germany), and the bone volume (BV; mm^3^), bone volume ratio (BV/TV), bone surface (BS; mm^2^), and bone surface/bone volume (BS/BV; mm^−1^) were calculated. After the micro-CT scan, we decalcified the specimens with 10% EDTA and soaked them in paraformaldehyde. After that, we selected the upper, middle, and lower sections of the specimens for HE, Masson, and Sirius red stain, respectively, as previously described [12, 19] (Fig. 1). The images were statistically analyzed using IPP software.

### Statistical analysis

All the presented data were expressed as the mean ± standard deviation. Statistical analysis was carried out by SPSS 14.0 for Windows. A group *t* test and one-way ANOVA single-factor analyses of variance (ANOVA) were used to compare values among groups. The significance level was set at *p* < 0.05.

## Results

### Construction of scaffolds

As shown in Fig. 1 (a–d), the PCL scaffolds, which was 3D-printed by FDM technology, had a diameter of 500 μm and a pore size of 1000 μm with a mode of 0–60–120° for easy access by osteogenic micro-tissue(see Fig. 1 (a–d)). Figure 1 (e) shows the morphology of calcium alginate gel.

### Construction of osteogenic micro-tissue

After culture for 5 days, the fetal rabbit BMSCs were adherently formed into cell colonies (Additional file 1: Figure S1A). The cells had shapes of “triangular” or “long fusiform.” By independent sample *t* test analysis, whose results were shown in Additional file 1: Figure S1B, we can see that BMSCs have a high potential of self-renewal and proliferation. After the P4 generation of BMSCs that were induced by osteogenic differentiation, many red positive nodules were observed under alizarin red staining, with a larger number and larger area (Additional file 1: Figure S1C). This result suggested that the osteogenic property of P4 BMSCs was good. After the P4 generation of BMSCs that were induced by adipogenic differentiation, lipid droplets in the cells were dyed red by Oil red O staining (Additional file 1: Figure S1D). This result suggested that P4 BMSCs had a good lipid performance. After the P4 generation of BMSCs that were induced by chondrogenic differentiation, highly aggregated cell mass was dyed blue, as clumps nodular or spiral shape, by Alcian blue staining (Additional file 1: Figure S1E).This result suggested that the P4 BMSCs had a good cartilage performance. From the above data, we can see that P4 BMSCs had the potential of multidirectional differentiation. BMSCs which were not induced could not be dyed.

After 2 weeks of osteogenesis, a white semitransparent membrane appeared at the bottom of the culture dish. Under light, the small sand-like plaques were evenly distributed, and occasionally part of the cell sheet edge was found curled. With cell scraper, the cell sheet could be gently stripped off from the dish (Additional file 2: Figure S2A). In the inverted microscope, the cells were dense growth, and the extracellular matrix was rich. The sand-like calcium deposits were around the cells, and many calcium nodules were formed (Additional file 2: Figure S2B). A lot of calcium nodules were dyed black by von Kossa staining in the cell sheet (Additional file 2: Figure S2C). By HE staining, the cells in the cell sheet were interwoven together, and the boundaries between cell and cell could not be observed (Additional file 2: Figure S2D-E). The nuclei were blue stained, and there was a large amount of extracellular matrix among the cells (Additional file 2: Figure S2D-E). The cell sheet was observed under a polarized light microscope, and it showed bright yellow, red, and green areas on it (Additional file 2: Figure S2F). It is suggested that the extracellular matrix of the cell sheet contained collagen I (bright yellow, red) and collagen III (green). On the other hand, the digested cells were stained by trypan blue and counted under the inverted microscope. The result was 1.5 × 10^7^/dish.

**Fig. 2:**
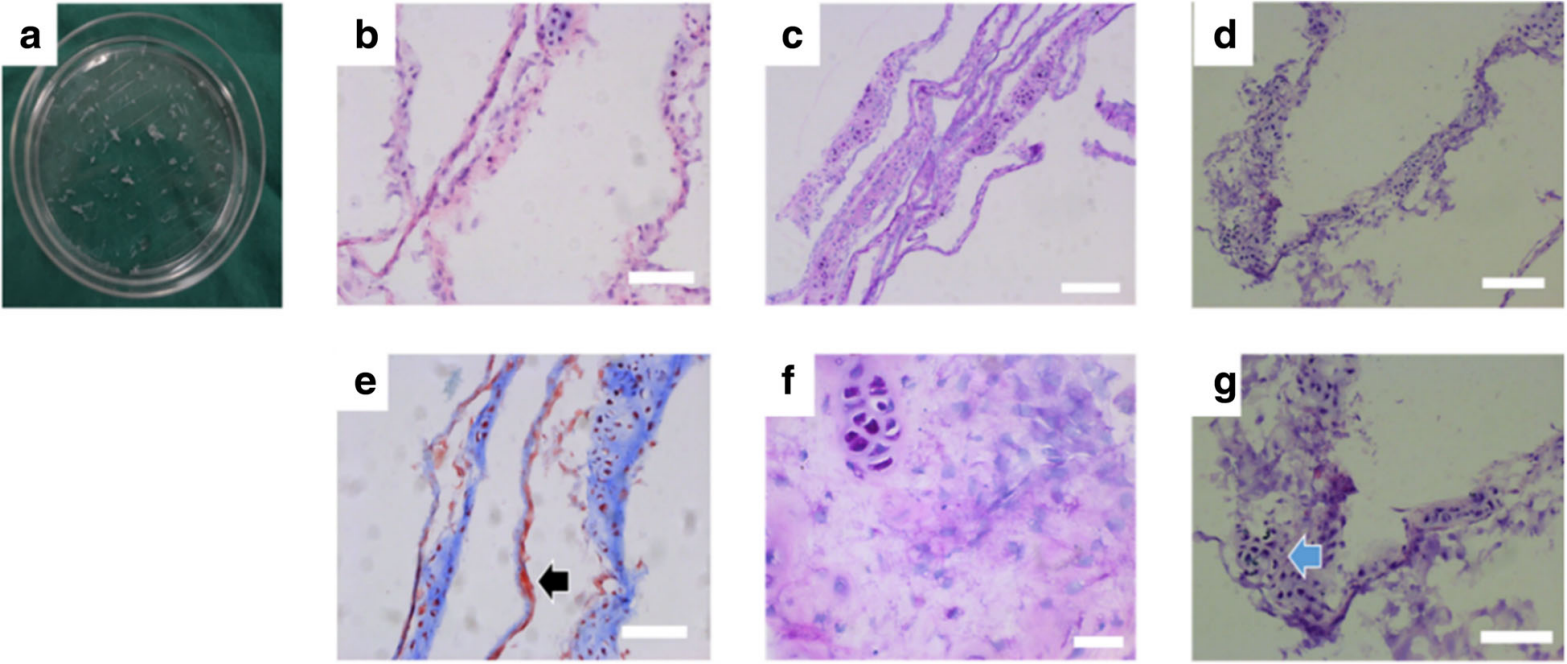
Osteogenic micro-tissue. **a** Fragmented cell sheet (generally). **b** HE staining (× 40). **c** PAS staining (× 40). **d** Von Kossa staining (× 40). **e** Masson staining (× 40, black arrow: extracellular matrix). **f** PAS staining (× 200). **g** Von Kossa staining (× 100, blue arrow, small calcium nodules). Bar: **c**, **d**50 μm; **b**, **e**, **g** 25 μm; **f** 10 μm

After the fragmentation of cell sheet, these tiny sheets could be self-crimped and folded (Fig. 2a). Each tiny sheet had a volume of about 0.5~0.8 mm^3^ and contained both cells and extracellular matrix, namely “osteogenic micro-tissue.” HE staining showed that the osteogenic micro-tissue was superposed membrane structure which was made by multilayer cells (Fig. 2b). Under the HE staining, the cells showed blue and the extracellular matrix showed pale pink (Fig. 2b). The cells in the cell sheet were closely interwoven together, and the boundaries between the cells could not be observed (Fig. 2b).Under Masson staining, the extracellular matrix showed blue or red (Fig. 2e). This result indicated that the extracellular matrix of the osteogenic micro-tissue was rich in collagen. Under PAS staining, the nucleus was dyed blue, and the extracellular matrix was purple red (Fig. 2c, f). It showed that the extracellular matrix contains rich glycogen. Under von Kossa staining, there were black or brown calcium salt deposits around the cells, which indicated that the extracellular matrix of the cells also contained small calcium nodules (Fig. 2d, g).

**Fig. 3:**
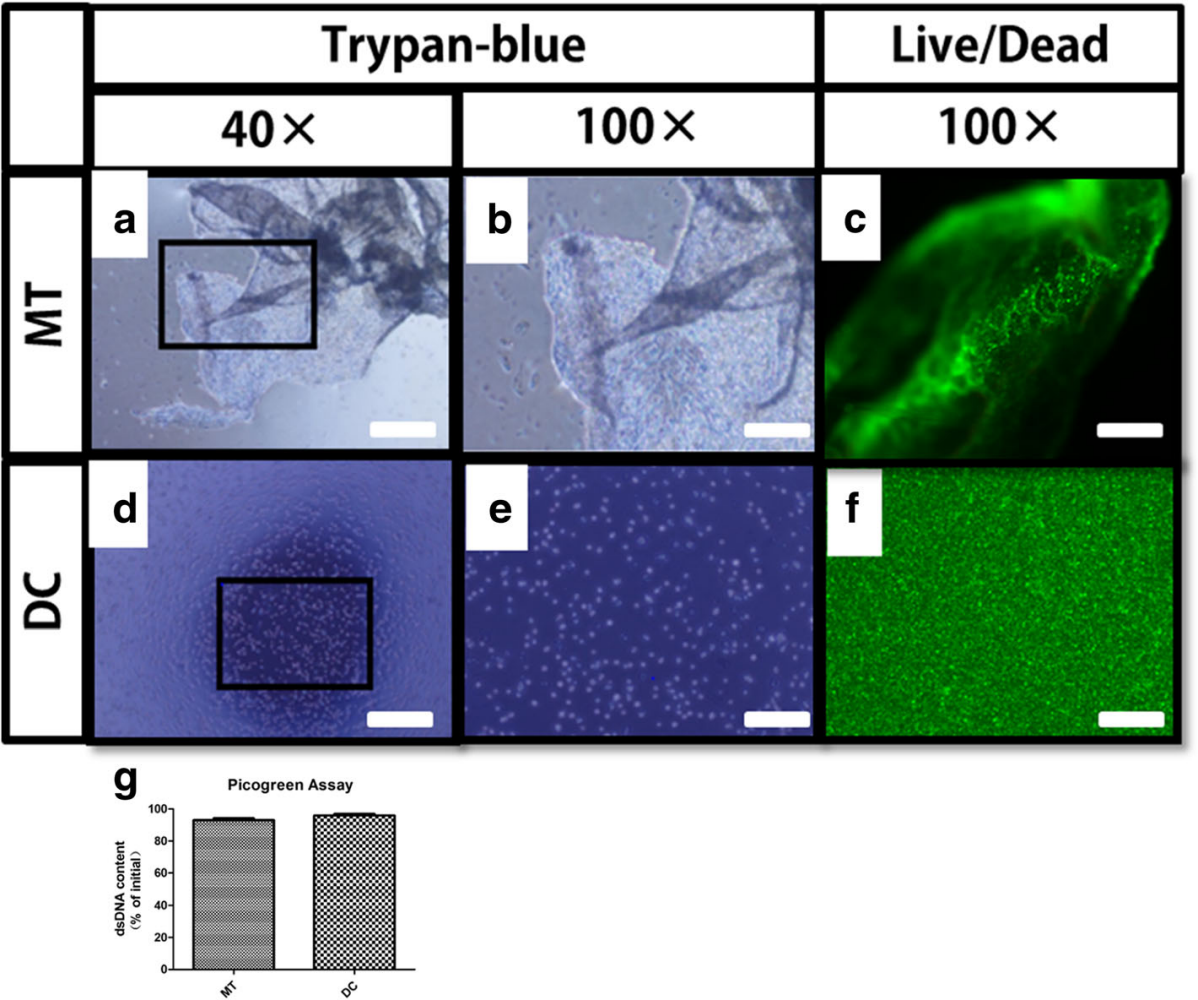
Comparison between osteogenic micro-tissue and digested cells in vitro. **a**, **b** Morphology of the osteogenic micro-tissue (**a** × 40, **b** × 100). **d**, **e** Morphology of the digested cell suspension (**d** × 40, **e** × 100). **c** Live/dead staining of the osteogenic micro-tissue (green color: live cells, red color: dead cells). **f** Live/dead staining of the digested cell suspension (green color: live cells, red color: dead cells). **g** Double-stranded DNA quantification of the osteogenic micro-tissue and digested cells. Scare bar: **a**, **d** 200 μm; **b**, **c**, **e**, **f** 80 μm. MT, osteogenic micro-tissue group; DC, digested cell group

Before the surgery, a comparison between osteogenic micro-tissue and digested cells in vitro was made. The micro-sheet folded structure of osteogenic micro-tissue was observed under the microscope after trypan blue staining (Fig. 3a, b). The control group using enzyme digestion, trypan blue staining showed that the cells were single-cell dispersion (Fig. 3d, e). Cell viability staining (Calcein-AM/PI) showed that all the digested cells obtained after fragmentation and enzyme digestion were stained green and no obvious dead cells were found, demonstrating that they all had cell viability after being treated (Fig. 3c,f). Picogreen assay showed that there was no significant difference (*p* > 0.1, *N* = 4) between the quantities of the double-stranded DNA of the osteogenic micro-tissue (93.00 ± 1.213%, *N* = 4) and the digested cells (95.90 ± 0.9958%, *N* = 4).Therefore, there was no difference between the cell numbers of the two groups.

### Osteogenesis-related proteins in osteogenic micro-tissue

**Fig. 4:**
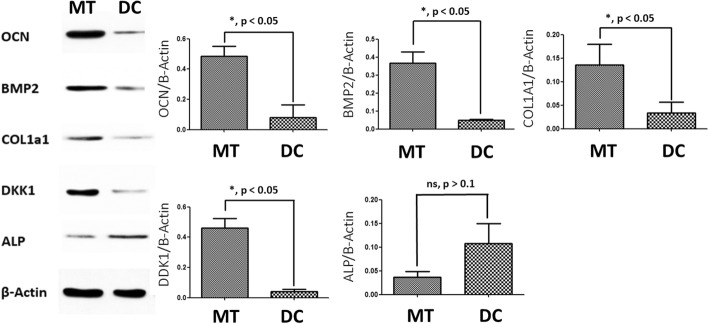
Comparison on osteogenic-related proteins (OCN, BMP2, COL1a1, DDk1, ALP) for osteogenic micro-tissue and digested cells by Western blot. MT, osteogenic micro-tissue; DC, digested cells

The same amount of osteogenic micro-tissue and digested cells (dishes) was extracted for Western blotting. Western blot was used to quantitatively analyze the amount of osteogenesis-related proteins such as OCN, BMP2, COL1a1, DKK1, and ALP in two groups. β-Actin was used as an internal control. The levels of OCN, BMP2, COL1a1, and DKK1 in the osteogenic micro-tissue group were significantly higher than those in the digested cell group (*p* < 0.05, *N* = 3), as shown in Fig. 4. However, there was no significant difference of the ALP level between the two groups (*p* > 0.1, *N* = 3).

### Repair the large segmental bone defects

#### Imaging examination and analysis

**Fig. 5:**
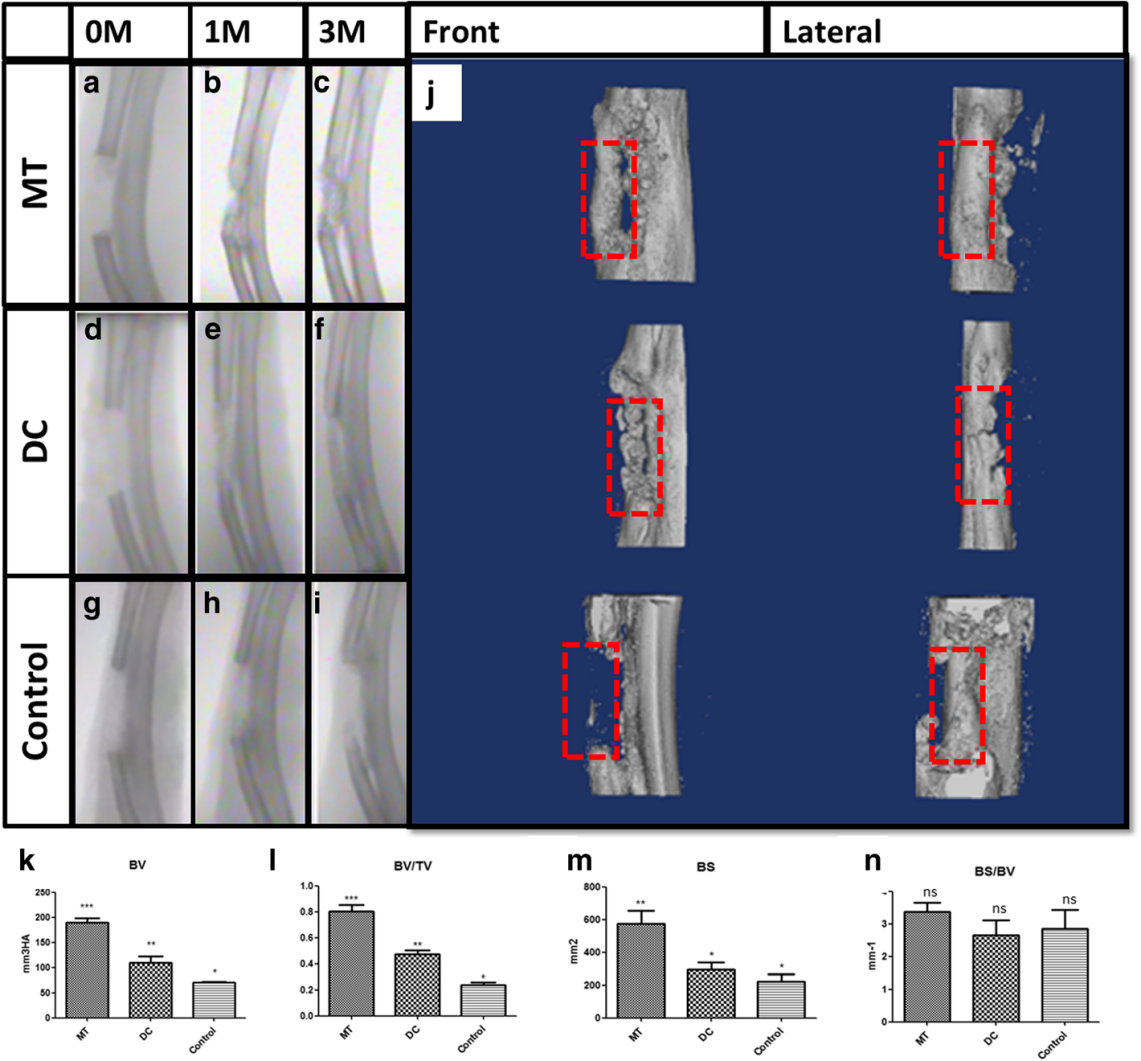
Imaging examination and analysis for in situ new bone formation. **a–i** X-ray comparison. **j** Micro-CT comparison. **k** Bone volume (BV) analysis of micro-CT. **l** Bone volume/tissue volume (BV/TV) analysis of micro-CT. **m** Bone surface (BS) analysis of micro-CT. **n** Bone surface/bone volume (BS/BV) analysis of micro-CT (mm^3^HA represents the volume of hydroxyapatite). MT, osteogenic micro-tissue; DC, digested cells; Control, control group

**Fig. 6:**
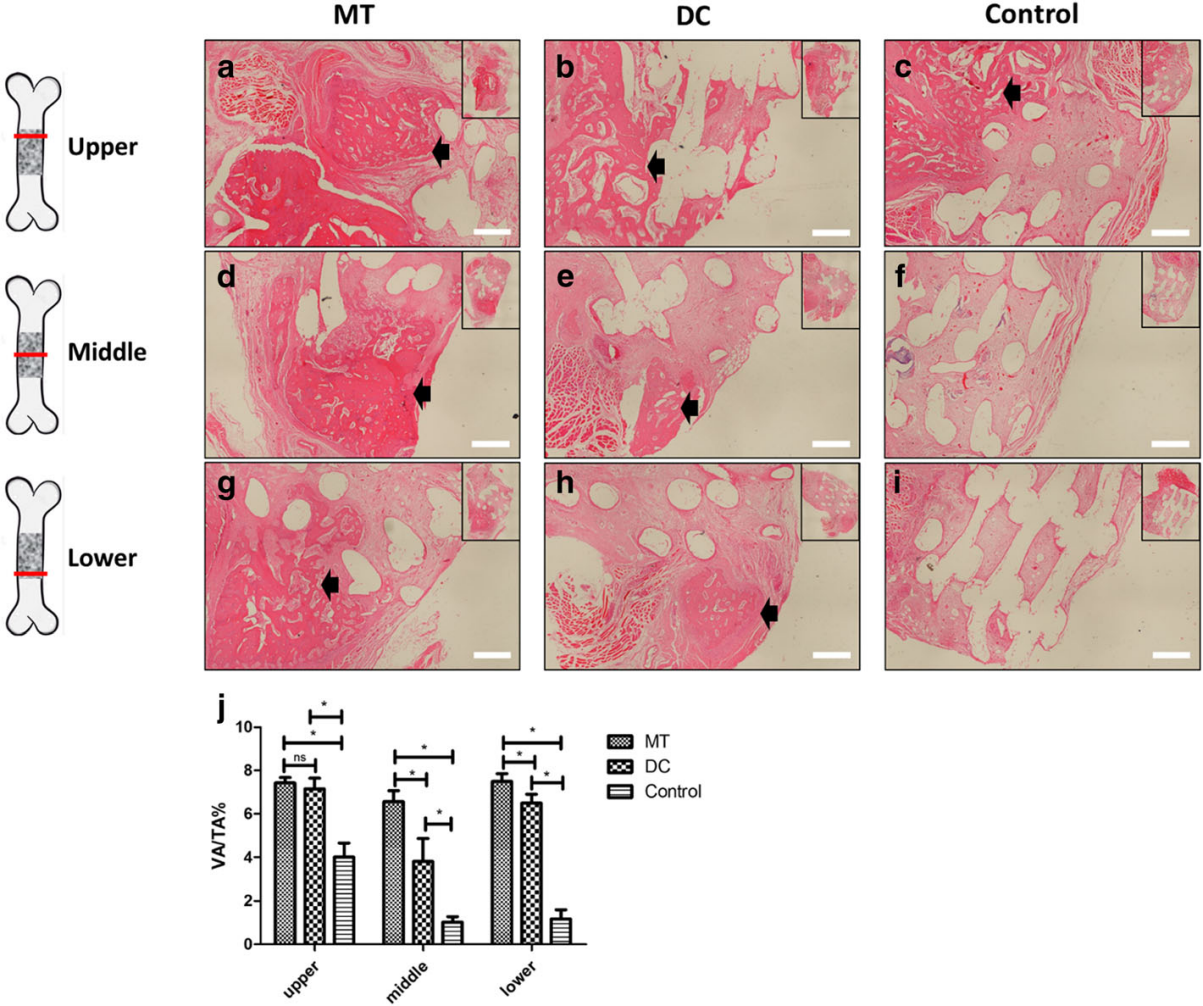
Comparison of HE staining for in situ osteogenesis of osteogenic micro-tissue and digested cells. In the upper, middle, and lower sections of the MT group (**a**, **d**, **g**), there was a significant bone-like structure (black arrow) in the scaffold. The bone-like structure was also found in the digested cell group (**b**, **e**, **h**), but the area was relatively small. In the blank control group (**c**, **f**, **i**), only a small amount of new bone was ingrowth into the upper part, and no obvious bone formation was observed in the other sections. **j** Vascularized area/tissue area (VA/TA, %) of the osteogenic micro-tissue, digested cells, control groups. The blank “round hole” is a space occupied by PCL scaffold (bar 500 μm). MT osteogenic micro-tissue, DC, digested cells; Control, control group

Immediately after surgery, after 1 month, and 3 months, respectively, the imaging examination was taken. After 1 month, new bone formation was seen in the bone defect area in the osteogenic micro-tissue group. After 3 months, there was a more complete shadow of the new bone formation, fully connecting the two ends of radius defects (Fig. 5a–c). The new bone formation could also be seen in the digested cell group after 1 month and 3 months (Fig. 5d–f). After 1 month and 3 months, in the control group, there was no significant new bone formation in the control scaffold (Fig. 5g–i). Comparing the osteogenic micro-tissue group, digested cell group, and the control group, the new bone formation shadow of the osteogenic micro-tissue group was more obvious. After euthanizing rabbits, the specimens were scanned by micro-CT and the data was analyzed and reconstructed by a computer (Additional file 3). The front and side faces of new bone in 3D images were compared. It can be seen that there were obvious new bone formation in the osteogenic micro-tissue group, which connected the two ends of the radius defect, and the new bone formation was obviously more complete than that in the digested cell group (Fig. 5j). In the control group, only small parts of new bone were formed at both ends of the bone defect, and they were far from connecting (Fig. 5j). As shown in Fig. 6k, the volume (BV) of bone formation in the osteogenic micro-tissue was 172.0 ± 18.59 mm^3^ (*N* = 4), that of the digested cells was 105.4 ± 9.426 mm^3^ (*N* = 4), and that of the blank control group was 70.26 ± 1.236 mm^3^ (*N* = 4). As shown in Fig. 5l, the bone volume/tissue volume (BV/TV) of bone formation in the osteogenic micro-tissue was 0.8051 ± 0.04941 (*N* = 4), that of the digested cells was 0.4731 ± 0.02941 (*N* = 4), and that of the blank control group was 0.2385 ± 0.02198 (*N* = 4). As shown in Fig. 5m, the bone surface (BS) of bone formation in the osteogenic micro-tissue was 575.2 ± 81.29 mm^2^ (*N* = 4), that of the digested cells was 293.1 ± 44.22 mm^2^ (*N* = 4), and that of the blank control group was 221.7 ± 45.92 mm^2^ (*N* = 4). As shown in Fig. 5n, the bone surface/bone volume (BS/BV) of bone formation in the osteogenic micro-tissue was 2.849 ± 0.5896 mm^−1^ (*N* = 4), that of the digested cells was 2.660 ± 0.4611 mm^−1^ (*N* = 4), and that of the blank control group was 3.383 ± 0.2738 mm^−1^ (*N* = 4). Statistics showed that the BV, BV/TV, and BS of osteogenic micro-tissue were significantly more than those of the digested cell group and control group (Fig. 5k–m, *p* < 0.05). There are no differences between the BS/BV of the three groups (Fig. 5n, *p* > 0.1). From the results (Fig. 5), it was found that the bone formation of the osteogenic micro-tissue repair strategy is more.

#### Histological examination

We selected specimens of the upper, middle, and lower sections of three histological sections for HE staining, Masson staining, and Sirius red staining.

Through HE staining, from Fig. 6, we can see that there was a very obvious bone tissue-like structure in the osteogenic micro-tissue scaffold material in the upper, middle, and lower sections (Fig. 6a, d, g), while the bone tissue-like structure was also seen in the digested cell group, but the area was relatively small (Fig. 6b, e, h). In the control group, only a little new bone was formed in the upper sections, and no bone formation was observed in other sections (Fig. 6c, f, i). The vascularized area/tissue area (VA/TA, %) of the osteogenic micro-tissue is 7.430 ± 0.2494% (upper), 6.565 ± 0.5047% (middle), and 7.495 ± 0.3607% (lower). The vascularized area/tissue area (VA/TA, %) of the digested cells is 7.153 ± 0.4896% (upper), 3.800 ± 1.064% (middle), and 6.498 ± 0.4066% (lower). The vascularized area/tissue area (VA/TA, %) of the control group is 4.000 ± 0.6554% (upper), 1.018 ± 0.2622% (middle), and 1.168 ± 0.4271% (lower). Statistics showed that the vascularized area/tissue area (VA/TA, %) of osteogenic micro-tissue was significantly more than that of the digested cell group and control group in the middle sections (Fig. 6j, *p* < 0.05), and the vascularized area of the osteogenic micro-tissue group was more homogeneous than that of the digested cell group (Additional file 4: Figure S4a-c).

**Fig. 7:**
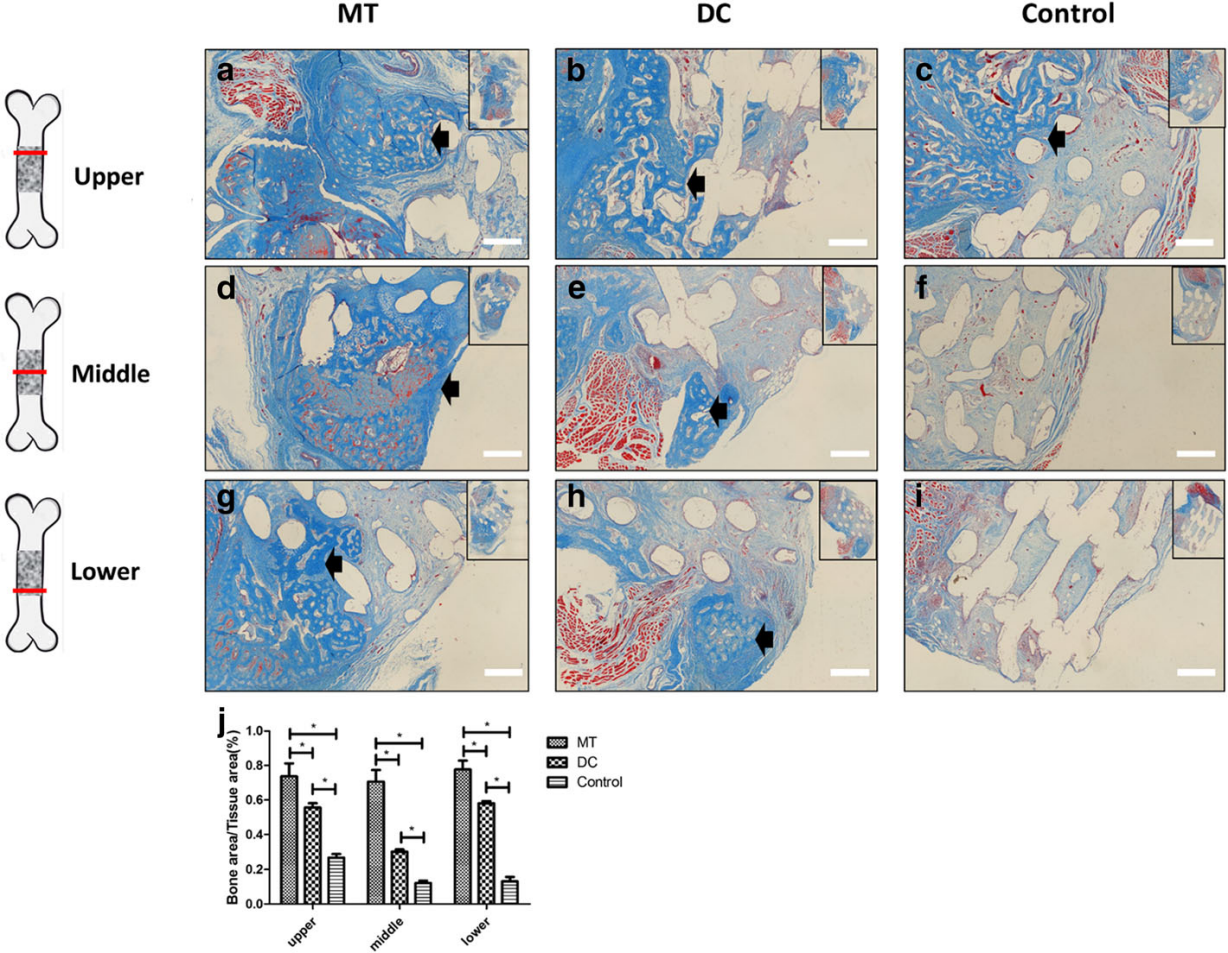
Comparison of Masson staining for in situ osteogenesis of osteogenic micro-tissue and digested cells. There was more bone-like structure in the micro-tissue group, which was ingrowth into the material, had more collagen (**a**, **d**, **g**, blank arrow) than digested cell group (**b**, **e**, **h**) and control group (**c**, **f**, **i**). **j** Bone area/tissue area (%) of the osteogenic micro-tissue, digested cells, control groups. The blank “round hole” is a space occupied by PCL scaffold (bar 500 μm). MT, osteogenic micro-tissue; DC, digested cells; Control, control group

By Masson staining, it is visible that there was no obvious bone collagen formation, but there was more fibrous tissue in the control group (Fig. 7c, f, i). In the osteogenic micro-tissue group, there was more blue bone tissue-like structure and relatively less fibrous tissue than in the digested cell group (Fig. 7a, b, d, e, g, h). The bone area/tissue area (%) of the osteogenic micro-tissue is 0.7369 ± 0.07500% (upper), 0.7066 ± 0.06727% (middle), and 0.7770 ± 0.05089% (lower). The bone area/tissue area (%) of the digested cell is 0.5565 ± 0.02441% (upper), 0.3006 ± 0.01338% (middle), and 0.5806 ± 0.01332% (lower). The bone area/tissue area (%) of the control group is 0.2660 ± 0.02151% (upper), 0.1210 ± 0.01333% (middle), and 0.1310 ± 0.02623% (lower). It indicated that the osteogenic micro-tissue group had more collagen growing into the scaffolds than the digested cell group (Fig. 7j, *p* < 0.05), and the bone collagen area of the osteogenic micro-tissue group was more homogeneous than the digested cell group (Additional file 4: Figure S4d-f).

**Fig. 8:**
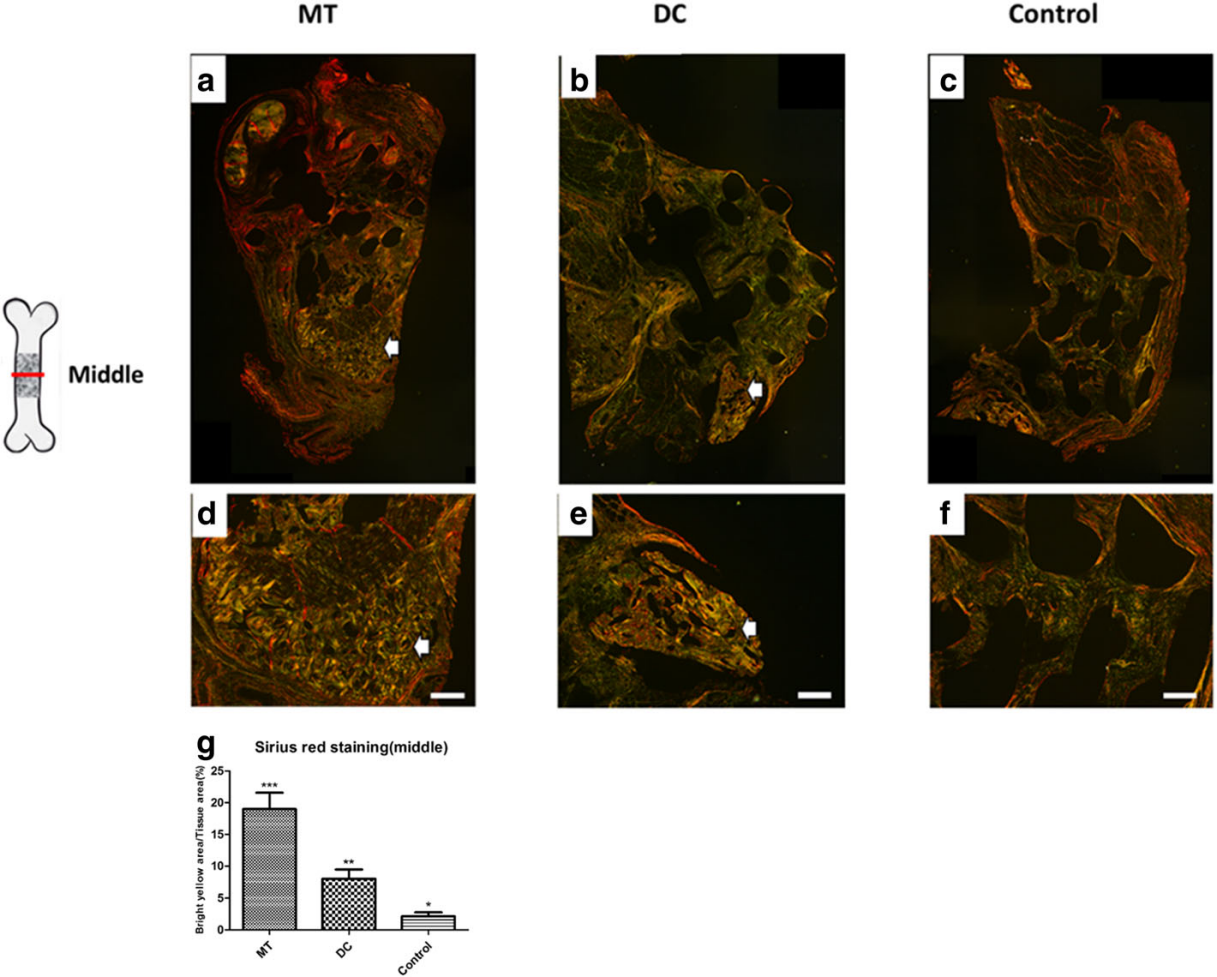
Picrosirius Red staining of the middle section of the three groups analyzed with polarization microscopy. **a**–**c** Panorama of the middle section of the three groups. **d**–**f** Pictures of the middle section of the three groups (× 40). **g** The osteogenic micro-tissue group had more collagen I (bright yellow, white arrow) (collagen I: bright yellow, strong light; collagen II: color grid, weak light; collagen III: green, thin fibers). Scale bar 500 μm. MT, osteogenic micro-tissue; DC, digested cells; Control, control group

Sirius red staining was carried out in the middle section. The bright yellow area/tissue area (%) of the osteogenic micro-tissue in the middle sections is 19.00 ± 2.582%. The bright yellow area/tissue area (%) of the digested cells is 8.000 ± 1.472%. The bright yellow area/tissue area (%) of the control group is 2.125 ± 0.6575%. The bright yellow area in the osteogenic micro-tissue group was more than the other two groups, indicating that the content of collagen I in the osteogenic micro-tissue group was more than that in the other groups, which showed that the collagen in osteogenic micro-tissue group was more mature (Fig. 8, *p* < 0.05). Therefore, by the osteogenic micro-tissue strategy, the new bone formation is more mature and more conducive in the repair of large bone defects.

## Discussion

In this study, we propose a new iBTE strategy for large segmental bone defect based on micro-tissue. We chose the “osteogenic micro-tissue,” which was obtained directly from the fragmentation of the cell sheets, in in situ iBTE strategy for the large bone defects. This method does not contain other material components except cells and extracellular matrix and can effectively avoid the impact of other material remnants, although its geometry is irregular [21]. In recent years, with the development of micro manufacturing technology, more technical methods have been applied to the construction of regenerative medicine and tissue engineering, including microfluidic technology, micro-carrier technology, microarray technology, and microgel technology [22–25]. These technologies are used to make micro-tissue, that is, to simulate the microstructure of natural tissues, and to fabricate an engineered tissue with tiny structure [22]. Although these various micro-tissues could have regular geometric shapes, they contain a large amount of material remnants and lack of naturally occurring osteogenic extracellular matrix. Therefore, it needs further research to compare the osteogenic micro-tissue used in this study and other micro-tissues in other studies.

This strategy has higher efficiency of cell usage than the traditional strategy. By consulting recent literature, we have found that the feasibility of the tissue engineering repair strategy, which directly injects MSCs into the rabbit radius defect, has been fully demonstrated [26–28]. In the literature, the amount of the direct injected mesenchymal stem cells is 4 × 10^6^–3 × 10^7^ per 15 mm defect of the rabbit radius [26–28]. In our study, the low cell number condition was chosen, in order to highlight the advantages and disadvantages of the two repair strategies based on osteogenic micro-tissue and digested cells. The cell amount was only 1/20 to 1/3 of the cell amount in the literature, that was 1.2 × 10^6^ per 15 mm defect (1.5 × 10^7^/mL, 80 μl). However, even under the condition of such low cell amount (1/20–1/3 in the literature), we found that the osteogenic micro-tissue group still had a strong osteogenic repair effect, which was significantly better than the digested cell group. Therefore, it is obvious that in the repair of large segmental bone defect in situ, osteogenic micro-tissue repair strategy could achieve a higher efficiency on cell usage.

Besides the undamaged cell activity due to no enzymatic digestion, we believe that the main reason for the better effect of osteogenic micro-tissue lies in the retention of more extracellular matrix in the osteogenic micro-tissue. Through the in vitro experiments in this study, we found that a large amount of extracellular matrix was exited in the osteogenic micro-tissue, and it contained a lot of active substances for osteogenesis. By Western blot experiment, we found that the contents of OCN, BMP2, COL1a, and DKK1 in osteogenic micro-tissue group were all significantly higher than those in digested cell group. This may be related to the retention of more extracellular matrix in osteogenic micro-tissue. OCN, BMP2, and COL1A are widely reported to play a strong role in promoting osteogenesis [29–31]. Although there have been some reports that declare DKK1 could inhibit the osteogenesis by inhibiting WNT pathway, it could promote the proliferation of MSCs. MSCs could produce multiple proteins during osteogenesis, some of which widely existed in the extracellular matrix.

Therefore, based on the data we had above, we proposed a hypothesis: The better repair effect of osteogenic micro-tissue may be partly due to the better osteogenic microenvironment provided by extracellular matrix. Behind this hypothesis lie two possible mechanisms: On the one hand, this extracellular matrix may preserve the transplanted cells better in the bone defect and protect them from the damage of early inflammatory factors. On the other hand, this extracellular matrix may contribute to the osteogenesis of the transplanted cells. If this hypothesis is established, this study also suggests that bone tissue engineering practice should follow the principle of preserving some of the extracellular matrix which may promote osteogenesis and avoiding the use of enzymes which would destroy the cell activity and extracellular matrix. More research about the effect of the ECM is needed in the future.

Although the endogenous changes, such as the endogenous cell migration or the inflammatory response of host tissues, might also affect the osteogenesis, this needs further studies.

## Conclusions

We have promoted a new iBTE strategy for large segmental bone defect based on osteogenic micro-tissue. It is confirmed that osteogenic micro-tissue can achieve a higher efficiency on cells usage and has a better repair effect than the digested cells. Although it needs further optimization, osteogenic micro-tissue repairing strategy would be a more promising clinical strategy to solve the problem of large segmental bone defect.

## Additional files

Additional file 1: **Figure S1.** Isolate, culture and identify the fetal BMSCs of rabbits. (a) fetal BMSCs obtained by the marrow cavity irrigation method; (b) the OD values of BMSCs (passage 4) on day 1, 3, 5, 7, 9; (c) Alizarin red S staining after 2 weeks of osteogenic induction of BMSCs (passage 4); (d) Oil red O staining after 3 weeks of lipid induction of BMSCs (passage 4); (e) Alice blue staining after 3 weeks of chondrogenic induction of BMSCs (passage 4); (f) Control, Alizarin red S staining after 2 weeks’ culture of BMSCs (passage 4); (g) Control, Oil red O staining after 3 weeks’ culture of BMSCs (passage 4); (h) Control, Alice blue staining after 3 weeks’ culture of BMSCs (passage 4). (i) Red area ratio (%) of the osteogenic inducted BMSCs and Control BMSCs after Alizarin red S staining. (j) Number of lipid droplets of the lipid inducted BMSCs and Control BMSCs after Oil red O staining. (k) Blue area ratio (%) of the chondrogenic inducted BMSCs and Control BMSCs after Alice blue staining. Scale bar: (a, c, e, f, h) 80 μm; (d, g) 20 μm. (BMP 6348 kb)

Additional file 2: **Figure S2.** Osteogenic cell sheets. A. osteogenesis cell sheets (general view); B. osteogenesis cell sheets (microscope observation); C. von Kossa staining of osteogenesis cell sheets; D. HE after staining osteogenesis cell sheets (40×); E. HE after osteogenesis cell sheets(100×); F. Sirius red staining of osteogenesis cell sheets, polarized light observation (40×).(Bar: B, C, D, F: 50 μm; E:20 μm) (BMP 3914 kb)

Additional file 3: **Figure S3.** Micro-CT examination and analysis for in situ new bone formation of all the specimens. (a-c) micro-CT comparison of the three groups; (d) bone surface (BS) analysis; (e) bone surface/bone volume (BS/BV) analysis; (f) bone volume (BV) analysis; (g) bone volume/tissue volume (BV/TV) analysis. (mm^3^HA represents the volume of hydroxyapatite). MT, osteogenic micro-tissue; DC, digested cells; Control, control group. (BMP 4622 kb)

Additional file 4: **Figure S4.** Vascularized area/tissue area (VA/TA, %) and bone area/tissue area (%) in three different sites of the osteogenic micro-tissue, digested cells, control group. The vascularized area and bone area/tissue area (%) of the osteogenic micro-tissue was more homogeneous than the digested cells group and control group. MT, osteogenic micro-tissue; DC, digested cells; Control, control group. (BMP 3614 kb)

## Abbreviations

BMSCs: Bone marrow stromal cells
DC: Digested cells
ECM: Extracellular matrix
FDM: Fused deposition modeling
iBTE: Injectable bone tissue engineering
MT: Osteogenic micro-tissue
PCL: Polycaprolactone
