# Supplementary figures and images for: Development of a micro-tissue-mediated injectable bone tissue engineering strategy for large segmental bone defect treatment
Source: Stem Cell Res Ther. 2018 Nov 28;9:331. doi: 10.1186/s13287-018-1064-1 (PMC6263540; doi:10.1186/s13287-018-1064-1)

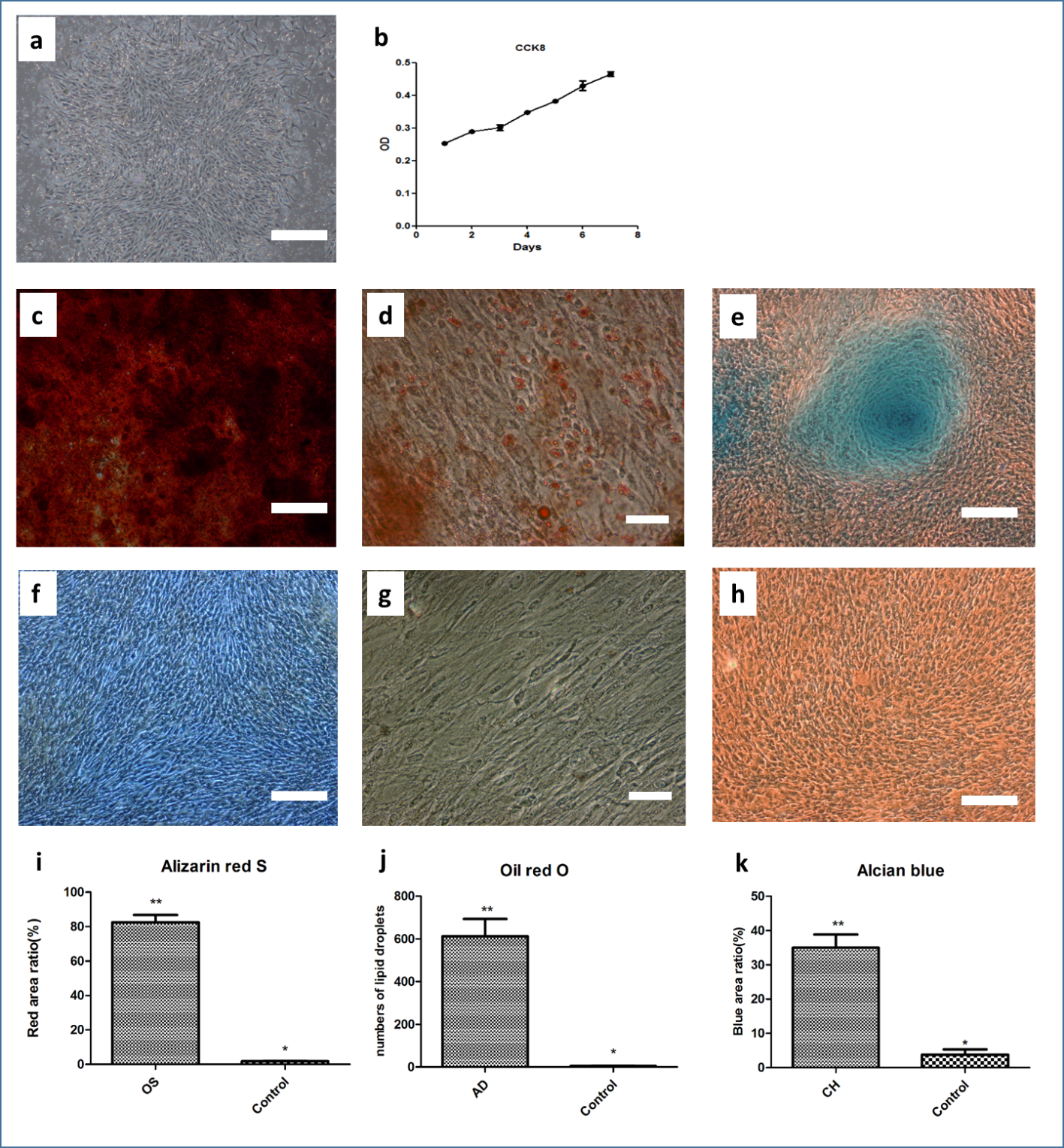

Supplement: Supplementary file 1 — Figure S1. Isolate, culture and identify the fetal BMSCs of rabbits. (a) fetal BMSCs obtained by the marrow cavity irrigation method; (b) the OD values of BMSCs (passage 4) on day 1, 3, 5, 7, 9; (c) Alizarin red S staining after 2 weeks of osteogenic induction of BMSCs (passage 4); (d) Oil red O staining after 3 weeks of lipid induction of BMSCs (passage 4); (e) Alice blue staining after 3 weeks of chondrogenic induction of BMSCs (passage 4); (f) Control, Alizarin red S staining after 2 weeks’ culture of BMSCs (passage 4); (g) Control, Oil red O staining after 3 weeks’ culture of BMSCs (passage 4); (h) Control, Alice blue staining after 3 weeks’ culture of BMSCs (passage 4). (i) Red area ratio (%) of the osteogenic inducted BMSCs and Control BMSCs after Alizarin red S staining. (j) Number of lipid droplets of the lipid inducted BMSCs and Control BMSCs after Oil red O staining. (k) Blue area ratio (%) of the chondrogenic inducted BMSCs and Control BMSCs after Alice blue staining. Scale bar: (a, c, e, f, h) 80 μm; (d, g) 20 μm. (BMP 6348 kb) [file 13287_2018_1064_MOESM1_ESM.bmp]

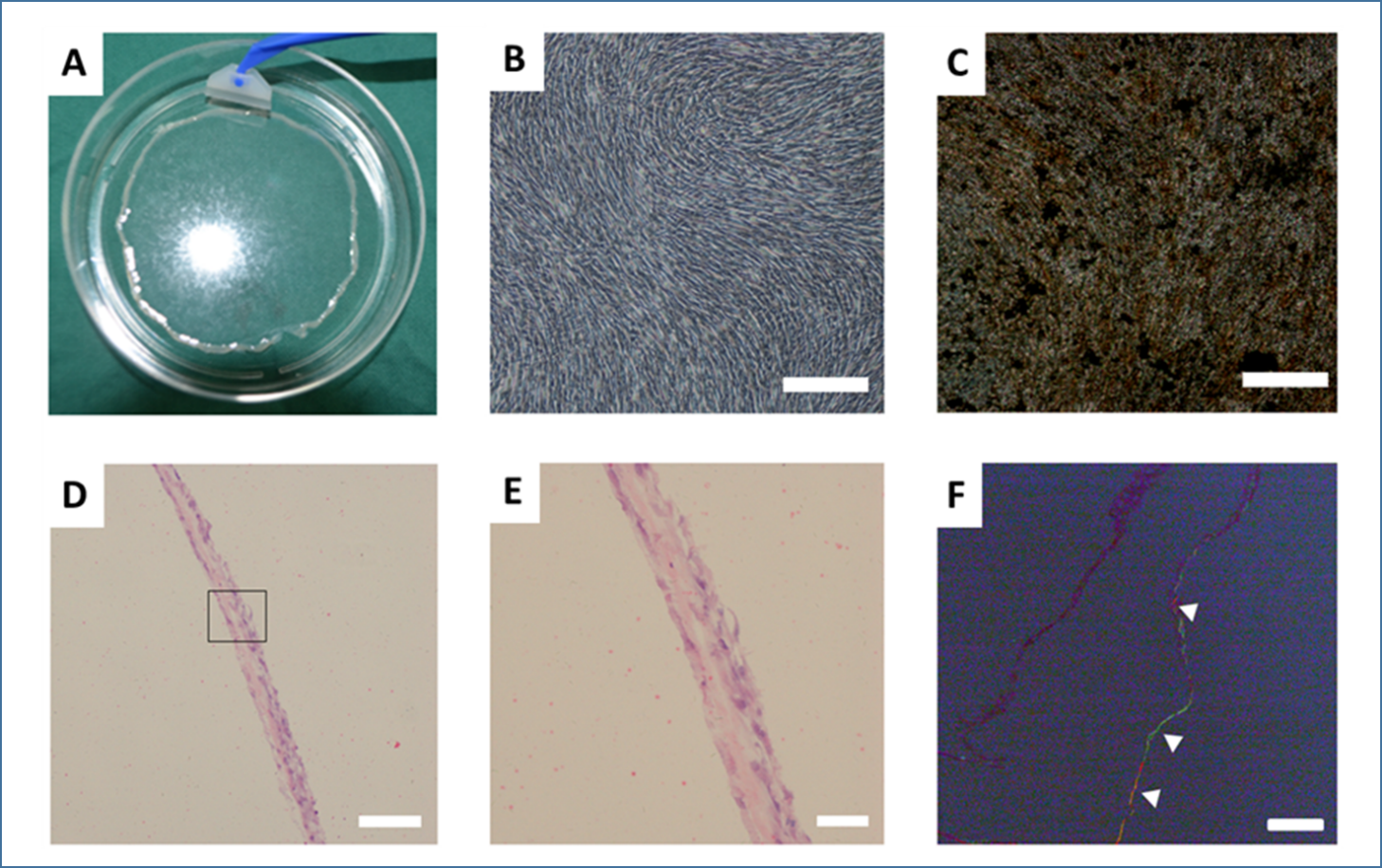

Supplement: Supplementary file 2 — Figure S2. Osteogenic cell sheets. A. osteogenesis cell sheets (general view); B. osteogenesis cell sheets (microscope observation); C. von Kossa staining of osteogenesis cell sheets; D. HE after staining osteogenesis cell sheets (40×); E. HE after osteogenesis cell sheets(100×); F. Sirius red staining of osteogenesis cell sheets, polarized light observation (40×).(Bar: B, C, D, F: 50 μm; E:20 μm) (BMP 3914 kb) [file 13287_2018_1064_MOESM2_ESM.bmp]

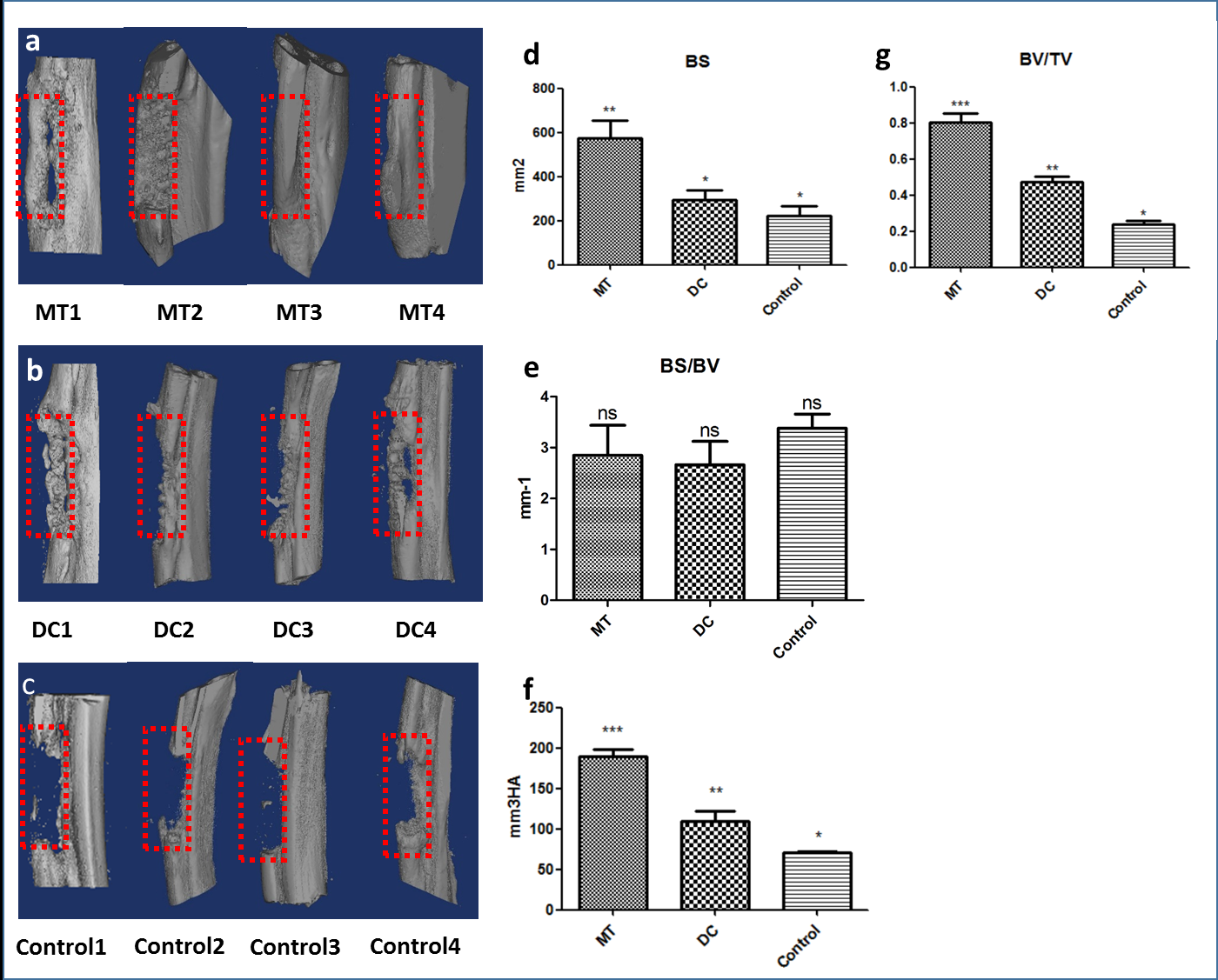

Supplement: Supplementary file 3 — Figure S3. Micro-CT examination and analysis for in situ new bone formation of all the specimens. (a-c) micro-CT comparison of the three groups; (d) bone surface (BS) analysis; (e) bone surface/bone volume (BS/BV) analysis; (f) bone volume (BV) analysis; (g) bone volume/tissue volume (BV/TV) analysis. (mm3HA represents the volume of hydroxyapatite). MT, osteogenic micro-tissue; DC, digested cells; Control, control group. (BMP 4622 kb) [file 13287_2018_1064_MOESM3_ESM.bmp]

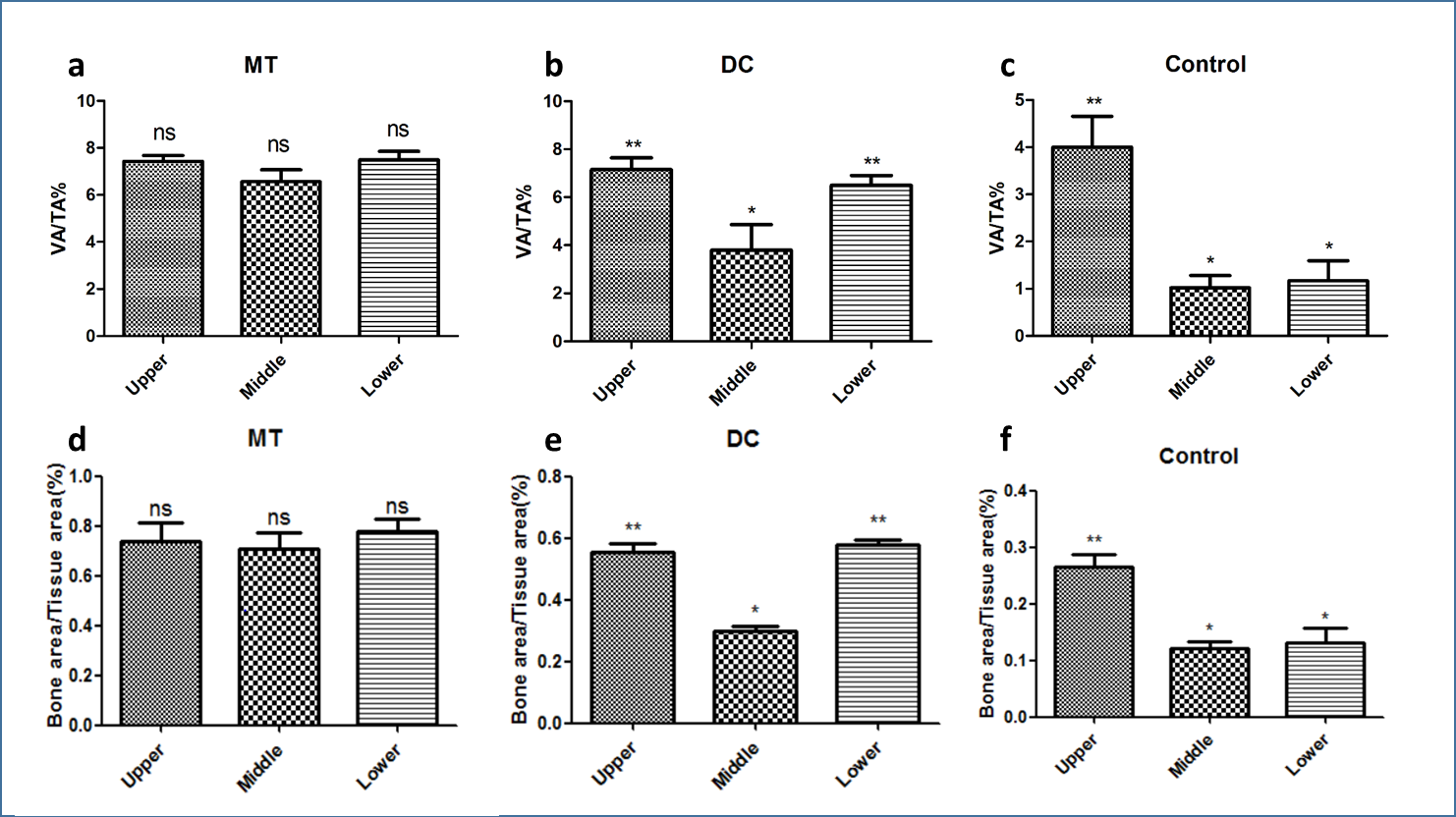

Supplement: Supplementary file 4 — Figure S4. Vascularized area/tissue area (VA/TA, %) and bone area/tissue area (%) in three different sites of the osteogenic micro-tissue, digested cells, control group. The vascularized area and bone area/tissue area (%) of the osteogenic micro-tissue was more homogeneous than the digested cells group and control group. MT, osteogenic micro-tissue; DC, digested cells; Control, control group. (BMP 3614 kb) [file 13287_2018_1064_MOESM4_ESM.bmp]
